# Calling for allied efforts to strengthen digital health literacy in Sweden: perspectives of policy makers

**DOI:** 10.1186/s12889-024-20174-9

**Published:** 2024-09-30

**Authors:** Karin Schölin Bywall, Therese Norgren, Beatrice Avagnina, Marta Pisano Gonzalez, Sarah Wamala Andersson, IDEAHL consortium

**Affiliations:** 1 School of Health, Care and Social Welfare, Division of Public Health Sciences, Mälardalen University, Västerås, Sweden; 2 Department of Public Health and Caring Sciences, Centre for Research Ethics & Bioethics, Uppsala University, Uppsala, Sweden; 3 Consulta Europa Projects and Innovation, Asturias, Spain; 4 General Directorate of Care and Socio-Health Care Coordination. Health Research Institute of the Principality of Asturias (ISPA), Oviedo, Asturias, Spain

**Keywords:** Digital health literacy, Policy makers, Digital health divide, EU-strategy

## Abstract

**Background:**

A more digitalised world comes with the promise to improve people’s lives. Therefore, it is essential that policymakers also align digital interventions with initiatives to empower citizens and strengthen their digital health literacy. The aim of this study was to explore the views of Swedish policymakers regarding the potential and barriers of a European strategy to strengthen digital health literacy.

**Method:**

Representatives from Swedish governmental agencies and regions were purposively approached by email to ask them to participate in online workshops to discuss the potential and barriers of developing a European strategy to strengthen digital health literacy.

**Results:**

The results highlight the need for a national strategy to strengthen digital health literacy. The findings point to critical areas for improvement, ethical and social considerations, and the importance of inclusive and accessible health information online. Participants identified vulnerable groups requiring targeted support to enhance their digital health literacy, particularly those at risk of digital exclusion. Participants emphasised the importance of considering various combinations of conditions or problems that people may have, urging policymakers to adopt a nuanced approach to enhance digital literacy.

**Conclusions:**

There is a critical need for policymakers to strengthen digital health literacy in the population to ensure health equity in relation to digitalisation opportunities. Policymakers advocate for a dedicated national strategy, supporting policymakers to prioritize digital health literacy. Tailoring information, enhancing digital support for prevention, and considering ethical implications are reported as important aspects to improve digital health literacy.

**Supplementary Information:**

The online version contains supplementary material available at 10.1186/s12889-024-20174-9.

## Background

Digitalisation has revolutionised the way people live, work, and interact with health-related information [1]. Digital health holds the potential to positively impact on individuals’ health and well-being through increased access to digital health technologies, information and virtual treatment [2, 3].

Using digital technology, individuals may access health information, track their health goals, and communicate with healthcare providers [4–6]. However, this transformative force in society raises questions about how best to use it while ensuring that individuals have the capacity to comprehend and benefit from it [7, 8]. In today’s rapidly evolving digital society, individuals’ digital health literacy plays a crucial role in influencing broader public health determinants [1, 9]. Digital health literacy is the ability to effectively read, write, and communicate about health using technology, evaluate the relevance and trustworthiness of health information online, and apply this information in various contexts. Improving digital health literacy empowers individuals to manage their health proactively and helps prevent health inequalities [10].

A key risk of this digital development in health care is the ‘digital health divide’, that is being created because of the uneven ability among individuals to assess and use available digital technologies [11, 12]. This means that millions of people in the world are still unable to benefit from available health and welfare technology [13]. This inequity needs to be addressed in policies so that the public can access and use digital health technology to increase their health [14, 15]. With an increasing emphasis on digital health literacy as a critical skill for citizen, professionals and organisations it is crucial to assess and understand the viewpoints and perspectives of those who formulate policies [16, 17].

Several ongoing initiatives in Europe aim to strengthen digital health literacy. Such as the Digital Health Europe (DHE) Project, funded by the EU’s Horizon 2020 program, supports digital health innovation and develops tools to improve digital health literacy (digitalhealtheurope.eu). Also, the eHealth Network than promotes interoperability of electronic health systems and aims to improve digital skills among healthcare professionals and citizens (health.ec.europa.eu). The European Digital Education Action Plan that addresses digital skills relevant to health literacy, emphasizing the importance of critically assessing online health information. An additional example is the Joint Action on Health Equity Europe (JAHEE), that focuses on reducing health inequalities by enhancing digital health literacy, including national strategies and training programs for vulnerable populations [18].

In Sweden, health literacy is a critical factor influencing public health outcomes, particularly among older adults and migrant populations [19]. Studies have shown that a significant proportion of older adults in Sweden have limited health literacy, which is influenced by factors such as age, educational level, vision, and cognitive ability [20]. This limited health literacy can lead to difficulties in understanding and using health information, negatively impacting health outcomes. For migrants, health literacy is a crucial factor for successful integration. Research indicates that culturally adapted health communication can improve health literacy and facilitate integration, which is particularly important in a multicultural society like Sweden [21].

This study was conducted within the framework of another EU project, the Improving Digital Empowerment for Active Healthy Living (IDEAHL) project, financed by the European Commission (101057477). The IDEAHL project, aimed at enhancing digital health literacy across Europe, finished in the development of the comprehensive European Digital Health Literacy Strategy in 2023 [22]. The IDEAHL project carried out co-creation workshops with stakeholders of digital health literacy (i.e., citizens, health care providers, researchers, policy makers, experts, and vulnerable groups) in 10 EU member states. Policy makers in Sweden were the focus of this study, as one of the representatives to the co-creation workshops. This study aimed to explore the views of Swedish policymakers regarding the potential and barriers of a European strategy to strengthen digital health literacy.

## Method

### Participants

Policymakers (i.e., representatives from governmental agencies and all Swedish regions) were invited to participate in 2 scheduled workshops that were built on each other. In Sweden, the country is divided into 21 administrative regions, each responsible for healthcare, regional public transportation, and cultural activities. These regions manage hospitals, public transport systems, and support local development, governed by elected regional councils. All the Swedish regions (*n* = 21) and government agencies working within the area of digital health literacy were invited to the workshops by email. The IDEAHL project included 14 EU partners. Co-creation workshops with stakeholders of digital health literacy (i.e., citizens, health care providers, researchers, policymakers, experts, and vulnerable groups) were carried out in 10 EU member states. This study reports the result from the workshop with policy makers in Sweden. The invitation to participate was send out via email, that included information about the IDEAHL project and the study. The participants and the researchers did not know each other prior to the inclusion of this study. All participants provided informed consent. The COnsolidated criteria for REporting Qualitative research (COREQ) checklist were considered during the report of the results (see supplementary material).

### Workshops with policy makers

Workshops with policy makers in Sweden aimed to answer to the research question; what are the potential and barriers of developing a European strategy to strengthen digital health literacy? The selection of method to conduct digital co-creation workshops was based on the availability of the participations that are working at different locations across Sweden to minimise travel time. The researchers did not know the participants before the invitation was send out. Additionally, the method selection was based on an assumption that the format of digital co-creation workshops using PowerPoint presentation to present the questions would support the participants to focus on the questions.

The online co-creation workshops were scheduled for 1 h each and hosted and recorded in Teams. We used an interview guide provided by the IDEAHL project. Minor framing of the initial questions in the interview guide was made to fit the target group of policy makers (see Table 1 for interview guide). In total, three workshops were conducted. The first workshop aimed to introduce the participants to the IDEAHL project and to collect information on participants experience and understanding of digital health literacy from a policy perspective to inform the audience about the IDEAHL project. In total, 8 participants contributed to the workshop.

The second workshop was initiated by presenting the findings from the first workshop and aiming to develop the discussion further and gain a better understanding of the results from workshop 1. The second workshop included questions to discuss the role of policy makers in strengthening digital health literacy for marginalised groups. The same 8 participants contributed to the second workshop. In addition to the main workshops, a third workshop was initiated to include those invited that were not able to attend the main ones. For the third workshop, 2 participants contributed. The workshops were conducted by two researchers. KSB, a female researcher in health and welfare technology at the time, with great experience in conducting qualitative research coordinated the workshops. KSB introduced the aim of the co-creation workshop, taking notes during the workshop and summarised the discussion in the end to confirm the highlights and made space for further clarifications. The workshops were moderated by TN, a research assistant at the time, with experience in qualitative interviews. TN made sure that all the participants were included in the discussion and all the questions were covered in the discussion.

**Table 1:** Interview guide for workshops 1 and 2

Workshop 1
Introduction to the research project and consent to participate
Start recording
Definition of digital health literacy
A round table introduction of participants
What are the barriers related to digital health literacy? • Health promotion • Disease prevention • Treatment • Self-care
What is needed to improve digital health literacy?
What kind of support is needed for different groups? • What groups needs extra support? • Examples of support intervention • How to account for this in the EU- strategy?
Intervention • Are you familiar with any intervention, policy, method, or programme for digital health literacy? • What can we bring to the EU-strategy?
Strengthen digital health literacy and ethical values: • Equality and justice • Autonomy • Integrity • Third part
Workshop 2
Summary of previous workshop to develop the discussion
There is a need for a national strategy to: • Strengthen digital health literacy? • What are the roles of governmental agencies and organisations? • How can they contribute to the EU strategy?
There are different obstacles for digital health literacy than for digital literacy and health literacy: • Can you develop the discussion? • Do you have any examples of specific obstacles related to digital health literacy? • How to consider that in the EU-strategy?
Different vulnerable groups need different support: • Which groups need extra support? • Do you have any examples of support intervention? • How to consider this in the EU strategy?
Intervention • Are you familiar with any intervention, policy, method, or programme for digital health literacy? • What can we bring to the EU-strategy?
Strengthen digital health literacy and ethical values: • Equality and justice • Autonomy • Integrity • Third part
Summary of discussion

### Analysis

The workshops lasted for 1 h each and was recorded, transcribed and analysed using qualitative content analysis by the authors KSB and TN. We applied qualitative content analysis methodology, a research method employed to systematically analyse text-based data in healthcare research [23]. It involves the identification and interpretation of patterns, themes, and meanings within the content of the data in a stepwise approach. Step (1) the researchers read through the transcripts of the focus groups to get an initial view of the content. Step (2) meaning units were selected and given a code. Step (3) the codes were refined and sorted into 3 overarching themes. The researchers continuously reviewed and refined the overarching themes to ensure they accurately represented the data. Throughout the analysis, the researchers strived to maintain a reflexive and iterative approach, by following an inductive approach [24]. The final list of codes and overarching themes were reviewed and refined by all authors. The analysis is presented in Table 2.

**Table 2:** Analysis

Meaning units	Codes	Overarching themes
Critical need for a national strategy to strengthen digital health literacy across Sweden	Need for alliance	Call for action to strengthen digital health literacy
Health information is not adapted to age, context and the individual’s/group’s level of digital health literacy.	Room for improvements	
Develop digital support for prevention and health promotion purposes	Target areas with potential	
Targeted support is needed to strengthen digital health literacy in individuals with a higher risk of digital exclusion	Targeted actions	Ethical and social aspects to acknowledge in a digital health literacy strategy
The importance of how to handle personal data is especially great for people with disabilities because of the history of mishandling such information.	Privacy matters	
Important to meet the individuals needs by assessing digital health literacy level of the targeted group	Meeting individuals needs	
The distribution of information online is essential to reach the targeted group	Information reach	Building bricks of a strategy to strengthen digital health literacy
It is important to understand that the target group for easy reading is so much larger than just people with intellectual disabilities	Accessibility of information	
Source criticism has never been so important as it is now	Support critical reflection	

## Results

In total, 10 policy makers participated in 3 focus groups. The participants included (*n* = 5) representatives for Swedish government agencies and representatives from Swedish regions (*n* = 5). The participants included both women (*n* = 6) and men (*n* = 4) in different ages. All the participants had professional experience from working with digital health literacy. The 2 pre-scheduled workshops included the same 8 participants. Additionally, 1 workshop was scheduled for 2 participants that were not able to attend the pre-scheduled.

The qualitative content analyses revealed the 3 main categories: (1) Call for action to strengthen digital health literacy, (2) Ethical and social aspects to acknowledge in a digital health literacy strategy, and (3) Building bricks of a strategy to strengthen digital health literacy.

### Call for action to strengthen digital health literacy

#### Need for alliance

Participants stressed that digital health literacy is not currently on the agenda for policy makers in Sweden to the same extend, as in other parts of Europe. Participants identified a critical need for a national strategy to strengthen digital health literacy across Sweden. They all agreed that governmental agencies in Sweden have limited knowledge about digital health literacy and how to work with digitalisation in health care. It was discussed that a national strategy could be developed in two different directions. Either as a separate strategy or a more integrated strategy aligning with already existing strategies and frameworks in Sweden. Here participants were more in favour of the integrated approach to add digital health literacy in existing frameworks on digitalisation and health care. One of the participants stressed that:

*Sweden is lagging in digitalisation compared to the Nordic countries and that Sweden needs a more basic infrastructure for digitalisation before setting a strategy for digital health literacy.*

The importance of separating and addressing interventions for health promotion and disease prevention using digital technologies was brought up for discussion. Participants stressed that the target groups level of digital health literacy always needs to be considered for digital health promotion and prevention interventions. One of the participants said that:

*Individuals’ digital health literacy can change over time*,* such as when a person gets older or ill*,* and then needs different interventions than others who do not have digital health literacy at all.*

Examples were given regarding courses and guidance that existed during the pandemic for booking an appointment for vaccination. Occupational health care was mentioned as a potential actor in working with digital health literacy because they meet people who do not seek medical care because they are sick.

#### Room for improvements

It was stressed that; the basic level of digital health literacy is not achieved in vulnerable groups. This may be because the health information is not adapted to age, context, and the individual’s/group’s level of digital health literacy.

Also, participants discussed, that the individual’s level of digital health literacy affects the ability to find the right platforms and information when searching. As well as the ability to review the credibility of the information based on the sender. Individuals with a low level of digital health literacy may have difficulty identifying appropriate keywords and therefore end up in the wrong place when searching. An obstacle to achieving a basic level of digital health literacy can be about a behavioural shift. That means that individuals start using the internet to search for health-promoting information instead of only searching for health information when they need care. The individual’s level of digital health literacy can also be hindered by analytical ability to navigate available information to find appropriate information. One participant raised that:

*The design of digital services can create obstacles for individuals ending up further away from the system because the algorithms control where the individual ends up in their search. It is a matter of financial resources to be visible on the Internet where companies out-compete government organizations.*

Additionally, it was raised that, there may be other barriers to digital health literacy at the societal level than to digital literacy and health literacy.

*An obstacle to digital health literacy may be the digital exclusion of the population*,* which means that 1 million adults in Sweden lack e-identification*,* which is a prerequisite for being able to seek care on the internet.*

#### Target areas with potential

According to the participants, there is great potential for improvement in the digital health promotion and prevention to strengthen digital health literacy by adapting the information based on the target group and problems, as well as developing digital support for prevention and health promotion purposes. Participants also expressed that there is great trust in digital technologies that can relieve care and generate profits. Improvement potential from the public is that the individual can get access to good digital technologies in everyday life that strengthens the ability to make independent decisions in health matters. According to the participants, development of digital technologies can be improved by starting from users’ needs and identifying these with evidence-based methods early in the process. Public actors can learn from private actors by targeting and reducing the supply of services. Better management of health data is also needed to utilize the full potential of the material that the individual collect.

### Ethical and social aspects to acknowledge in a digital health literacy strategy

#### Targeted actions

Participants identified some specific groups in need for targeted support in strengthening levels of digital health literacy, those were individuals with a higher risk of digital exclusion (or digital divide). Examples include persons with intellectual disabilities, who need extra support in understanding and using digital technologies in health care. Also, young people that might not be so evolved in source criticism or in using different kinds of digital technologies, even though they are good at using internet or apps. However, one of the participants highlighted that it is important to think about individuals’ unique obstacles and attributes rather that categorizing individuals in certain groups. According to one participant:

*Policy makers need to ‘Keep in mind that people have different combinations of conditions or problems*,* and it may be better to think based on these parameters.*

It was also discussed that parents’ digital health literacy impact children’s levels of digital health literacy in several ways, partly through the parents’ own health/ill health. To promote children’s health, several actors in society such as employers and social insurance agencies need to take part in the interventions that promote health.

#### Privacy matters

Privacy was discussed in relation to digital technologies. It is difficult to maintain your private life if you cannot access information about your own health but needs help from others, for example if you have visual impairment. This also applies to the new state of digital identification “personal eID” and to be used by everyone. The vulnerable groups who have difficulty in using it, may not maintain their privacy regarding health issues when they need support from another person to use it. One of the participants said:

*It’s like*,* I would never have a person from the municipality who gets paid on my gynecologist visit*,* for example. After all*,* people living with disabilities are forced to have occasional or other types of doctors’ visit.*

The importance of how we handle personal data is especially sensitive for people with disabilities. However, it is also a dilemma that makes it difficult to obtain statistics for example on how many people need specific services from certain authorities, etc.

Participants discussed that GDPR was aimed at protecting privacy, but then a lot of responsibility falls on the individual to decide to, for example, accept all cookies or only necessary and understand what it means to do this or that. This also applies to public websites and the importance of taking responsibility for explaining what it is we collect, what is it we want to know about you and why.

#### Meeting individuals´ needs

One participant highlighted that the society wants to protect certain groups (such as young people with cognitive disabilities) from searching for information themselves:

*It’s a very important group that you don’t forget that society has unfortunately forgotten historically and probably*,* I think too*,* but they need extra support in understanding of course.*

It was also demonstrated that; prejudices can play a role in how information is designed for different groups. The individuals become their disability, so within the group of people with visual impairments there are people who have a doctorate, just as there are people who have completed high school.

### Building bricks of a strategy to strengthen digital health literacy

#### Information reach

The participants stressed that the distribution of information online is essential, i.e., how the information easily reaches the people you want to reach. If you want to reach out with health promotion measures, for example, it is good to take advantage of the type of technology available, such as push notifications. Or inform via regular mail with information about a good website:

*We think that’s why it’s digital content*,* we just have to use digital channels. But it actually works quite well to send out an analog mailing and say*,* look here.*

#### Accessibility of information

The discussion centred around enhancing accessibility of health-related information online. Participants stressed the importance of providing information in adaptable formats, facilitating easy comprehension. They emphasized the need for municipalities to organize and allocate resources for tailoring communication to the specific needs of the target audience. A key aspect highlighted was making the information personally relevant, especially for people with disabilities. The potential risk of delays in conveying information in easy-to-read formats was noted. The discussion expanded to encompass a broad audience, including those new to the language, with limited reading abilities, dyslexia, or hearing impairments. Participants presented examples illustrating the difficulty faced with existing health websites, underlining the urgency for more accessible content. During urgent situations, participants recommended simplifying language for effective communication. The role of caregivers as intermediaries and the necessity of optimizing mobile communication were acknowledged. Lastly, the conversation advocated for a universal design approach across all interventions to ensure greater accessibility for diverse users.

#### Support critical reflection

One aspect that was highlighted is credibility and credible sources. Participants stated that source criticism has never been so important as it is now. Many people have difficulty absorbing a text or understanding a text. Not being able to critically reflect on health-related information online can make people vulnerable to misinformation or disinformation. Participants stressed the schools´ responsibility to also equip young people to cope with digitization when we digitize, for example for source criticism.

*To theorize and learn more about these perspectives in teaching*,* it would make a difference*,* I think*,* to practice critical source skills or linguistic skills or something like that in an effort later in life is a big challenge*,* I think just choosing perspectives to strengthen the general lack of health literacy.*

## Discussion

The aim of this study was to explore the views of Swedish policymakers regarding the potential and barriers of a European strategy to strengthen digital health literacy. Results from engaging policymakers in co-creation workshops and analysing their insights in this study highlight critical aspects, such as the need for a national strategy to strengthen digital health literacy, potential target areas for improvement, ethical and social considerations, and the importance of inclusive and accessible health-related information online.

The goal is to inform policy decisions and contribute to shaping an effective European strategy that optimizes the societal benefits of digitalisation in the realm of healthcare. A need that is also stressed in previous research, highlighting the need for coordinated, multidisciplinary approaches and strategies for regulating, evaluating, and using digital technologies [25].

Policymakers clearly highlighted specific groups that require targeted support to enhance their digital health literacy, focusing on those facing a higher risk of digital exclusion or experiencing a digital divide. Among these groups are individuals with intellectual disabilities, who need additional assistance in comprehending and effectively utilizing digital healthcare technologies.

Another group are the young people who were identified to lack advanced skills in information source criticism and utilizing various digital tools, despite their frequent internet or app usage. These findings are in line with clinical implications for low or inadequate health literacy, stating that clinicians need to modify their communication with patients in response to lower levels of health literacy [26]. In addition to the relevance of digital health as a super determinant of health [6].

However, it’s essential to recognize that individuals possess diverse obstacles and attributes, making it crucial to tailor support based on individual circumstances and preferences rather than placing them into rigid categories. Participants emphasized the importance of considering various combinations of conditions or problems that people may have, urging policymakers to adopt a nuanced approach towards enhancing digital literacy.

One of the strengths of this study was the active engagement of policymakers as key informants in research.

This study has limitations, only 10 participants may not reflect the views of all policymakers in Sweden. Therefore, the results and conclusions should be interpretated as the views of the ones that participated and not to be the general view of policy makers in Sweden.

It can be argued that 10 are few, however the invite to participate was send out to all governmental agencies working with questions related to digital health literacy and all region in Sweden and the most appropriate representatives working with digital health literacy matters from the national agencies participated according to them. To strengthen the validity, we conducted one additional workshop with those that could not attend the main workshops. This study only included results from policy makers in Sweden, therefore making conclusions based on the results may not be transferable to all European countries. However, the co-creation workshops with stakeholders of digital health literacy (i.e., citizens, health care providers, researchers, policymakers, experts, and vulnerable groups) were carried out in 10 EU member states. In total the IDEAHL strategy includes 140 co-creation sessions with more than 1,400 stakeholders.

Trustworthiness is a key challenge in qualitative research. Therefore, three concepts were assessed in this study: credibility, transferability, and dependability [23]. Creditability was promoted in this study by inviting participants from all 21 regions and relevant agencies. Participants were informed about the IDEAHL project and the study before the workshop started, and they were given an opportunity to ask questions to the researchers (KSB and TN). Dependability was promoted in the analysis of the results by collaboration between the authors.

In summary, this study emphasizes the urgent need for a national strategy in Sweden to improve digital health literacy in Sweden and aligning with existing initiatives, customizing information, focusing on prevention, and those addressing ethical considerations. Research related to digital health literacy as a determinant of health is needed to inform policies and practice aiming at reducing inequalities. Addressing evidence related to digital health literacy is crucial in optimising the true value and effectiveness of digitalisation in enhancing health outcomes and population health.

## Conclusions

Digital technologies hold immense potential for improving public health, they also have the power to widen existing inequalities. Therefore, developing national policies and EU-strategies to strengthen digital health literacy are essential for tackling inequalities related to digitalisation and enhancing population health.

## Supplementary information

Supplementary Material 1.

## Data Availability

The datasets used and/or analysed during the current study available from the corresponding author on reasonable request.

## Data availability

No datasets were generated or analysed during the current study.
